# Substantial non-compliance of online pharmacy catalogues with guidelines

**DOI:** 10.1007/s00210-024-03571-0

**Published:** 2024-11-12

**Authors:** Lara Barlage, Roland Seifert

**Affiliations:** Institute of Pharmacology, Hannover Medical School, D-30625 Hannover, Germany

**Keywords:** Online pharmacy, Drug advertising, Advertisement, Pharmacy review, Guideline review, Self-medication, Product catalogue, Pubmed.gov

## Abstract

Online pharmacies are popular worldwide. In Germany, there were 150 active online pharmacies in 2022. As the competition is therefore fierce and new customers need to be acquired, some of these online pharmacies create catalogues that advertise products in self-medication to attract the interest of potential customers. This study analyzed the allergy, cold, and gastrointestinal tract categories from the summer/spring 2023 catalogues of two different online pharmacies in Germany. A comparison of the active ingredients in both catalogues and an analysis of the product’s conformity with the German AWMF (Association of the Scientific Medical Societies in Germany) guidelines as well as international guidelines were carried out. In medicine, guidelines serve as evidence-based decision-making aids tools to ensure adequate patient care. The online catalogue of online pharmacy 1 showed conformity of 65% with AWMF guidelines, and the online catalogue of online pharmacy 2 showed conformity of 59% with AWMF guidelines. The online catalogue of online pharmacy 1 showed conformity of 44% with international guidelines, and the online catalogue of online pharmacy 2 showed conformity of just only 17% with international guidelines. Thus, there is a substantial lack of conformity of online pharmacy self-medication catalogues with national and international guidelines. For several indication areas including market-relevant rhinosinusitis and irritable bowel syndrome, consensus between AWMF and international guidelines was poor. The lack of evidence-based presentation of online pharmacy catalogues constitutes a risk for consumer safety. This risk must be assessed in future studies, for example by studies on intoxications caused by self-medication that is non-compliant with guidelines. Our results also underline the necessity of a stricter surveillance of the German self-medication market.

**Supplementary Information:**

The online version contains supplementary material available at 10.1007/s00210-024-03571-0.

## Introduction

In medicine, guidelines are used as an orientation aid to make an adequate decision on how to proceed in the patient’s situation and to find the best personalized pharmacotherapy (https://www.awmf.org/leitlinien; last accessed 23 August 2024). Guidelines have become an important tool for physicians and health-related institutions (Nothacker et al. 2014). They set out recommendations that have been evaluated as the best treatment option available by assessing the current state of knowledge, conducting a harm-benefit analysis and exchanging opinions (Ollenschläger et al. 2000). Consequently, the guidelines enable physicians to treat their patients in accordance with evidence- and/or consensus-based recommendations (Voigt et al. 2023). The implementation of guidelines is intended to ensure quality assurance in patient care by guaranteeing a uniform and scientifically based approach (Ollenschläger et al. 2000). Guideline recommendations should not be understood as mandatory, but it is always necessary to check the applicability to the individual patient context and to adapt the guideline recommendation to offer the patient the best treatment available (https://www.awmf.org/leitlinien; last accessed 23 July 2024).

In Germany, the Association of the Scientific Medical Societies in Germany (AWMF, Arbeitsgemeinschaft der Wissenschaftlichen Medizinischen Fachgesellschaften e.V.) has been coordinating the development of guidelines on various indications by specialist societies since 1995, resulting in a broad registry of guidelines (https://www.awmf.org/die-awmf; last accessed 23 July 2024). The AWMF is an association of 183 medical societies, which aims to improve cooperation (https://www.awmf.org/die-awmf; last accessed 23 July 2024).

A retrospective study on the effectiveness of the implementation of guidelines was published in 2010, which dealt with the S3 guideline on breast cancer (Wöckel et al. 2010). Guideline-compliant treatment can improve the overall survival and recurrence-free survival of patients with breast cancer (Wöckel et al. 2010). A prospective controlled study showed that the implementation of the S3 guideline for the treatment of acute perioperative and post-traumatic pain through a certification measure led to an improvement in areas such as maximum and exertional pain and pain-related mobility and was superior to other quality improvement projects (Lehmkuhl et al. 2011).

Online pharmacies in Germany are permitted to ship over-the-counter (OTC) and prescription drugs, but they are subject to the same regulations as brick-and-mortar pharmacies and must be connected to a brick-and-mortar pharmacy (https://www.abda.de/themen/versorgungsfragen/versandhandel/; last accessed 16 October 2024). In addition, it is also possible for international online pharmacies to send drugs to Germany, if only drugs that are approved in Germany are sold (https://www.bundesgesundheitsministerium.de/themen/krankenversicherung/online-ratgeber-krankenversicherung/arznei-heil-und-hilfsmittel/apotheken; last accessed 15 October 2024). However, ordering and shipping drugs online involves risks for the consumer. On the one hand, it is not always obvious whether the website is a reputable online pharmacy and to make this easier, there is a European mail-order logo, which ensures that the provider is permitted to offer mail-order medicines under national law (https://www.bundesgesundheitsministerium.de/themen/krankenversicherung/online-ratgeber-krankenversicherung/arznei-heil-und-hilfsmittel/apotheken; last accessed 15 October 2024). On the other hand, there is a possibility of complications during the shipping process, such as the disruption of the cold chain for products that require refrigeration (https://www.abda.de/themen/versorgungsfragen/versandhandel/; last accessed 16 October 2024).

This paper analyzes the spring/summer product catalogue of two online pharmacies (OP) from 2023 for conformity of the products offered with national AWMF guidelines and international guidelines for nine indications. There were 150 active online pharmacies in Germany in 2022 (ABDA 2023). Some of them publish product catalogues to attract customers and advertise a selection of their product range. National and international mail-order sales of OTC drugs amounted to 1572 million euros in 2022, which corresponds to a market share of 20.7%, meaning that one in five OTC products is purchased via mail order (ABDA 2023). On average, 62% of the German population regularly purchased products from online pharmacies in 2021 (https://de.statista.com/statistik/daten/studie/901431/umfrage/online-kauf-von-medikamenten-in-deutschland-nach-alter/; last accessed 6 March 2024). This underlines the importance of online pharmacies and shows that they are indispensable in our digital age.

To date, there are no studies in the literature that examine products from the range of brick-and-mortar or online pharmacies for conformity with medical guidelines. Therefore, the aim of this study is to fill this research gap by examining the products offered for self-medication in the product catalogues of two online pharmacies for conformity with national and international guidelines. This is done exemplary for nine indications from the categories allergy, common cold, and gastrointestinal tract.

## Material and methods

### Online pharmacies and their product catalogue

In a recent study, we analyzed the compliance of the online catalogues of online pharmacies 1 and 2 (OP1 and OP2) with the Therapeutic Products Advertising Act (Barlage and Seifert 2024). To decide on two online pharmacies, we first identified online pharmacies that offer product catalogues. Then, we decided to select the top-selling online pharmacy that offers a product catalogue which is denoted as online pharmacy 1 (OP1) in this paper. In 2023, the top 15 online pharmacies in Germany generated total net sales of 2,830 million euros, of which over 19%, 550 million euros, were generated by OP1 (Sempora Consulting GmbH 2024). In contrast, we selected a smaller online pharmacy, which is referenced below as online pharmacy (OP2) and generated net sales of 85 million euros in 2021, which is 3% of the sales of the top 15 online pharmacies (Sempora Consulting GmbH 2024).

**Fig. 1:**
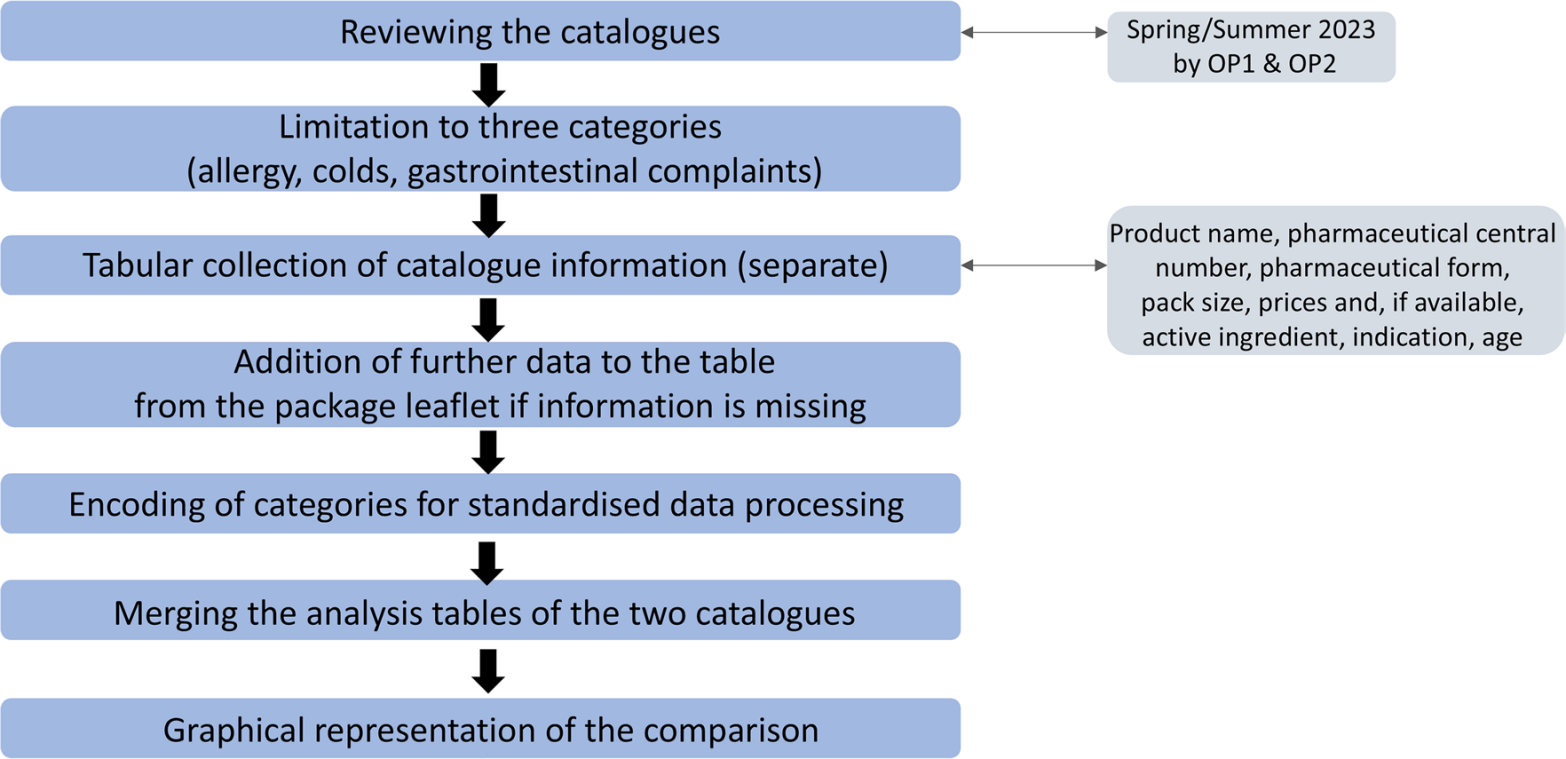
Graphical representation of the methods for comparing the product catalogues in a flow chart

The current analysis is based on the spring/summer 2023 product catalogues of the online pharmacies OP1 and OP2. Figure 1 shows the methodological procedure for catalogue analysis in this study. Both online catalogues of OP1 and OP2 are freely available online and could be requested free of charge as a paper catalogue from the online pharmacies during the period of validity. The catalogues present and advertise a selected part of the product range of both pharmacies for self-medication and are divided into areas of symptoms. The product catalogue of OP1 consists of a total of 70 pages including cover and back, and the catalogue of OP2 comprises a total of 40 pages.

This analysis is limited to products from the allergy, cold, and gastrointestinal tract areas. This means that 175 products on 11 pages from the OP1 product catalogue were included as well as 109 products on eight pages from the OP2 product catalogue. To select the three categories, we used the top ten list of the top-selling indication groups for OTC drugs from the report by the German Association of Pharmaceutical Manufacturers (Bundesverband der Arzneimittel-Hersteller e.V.) for 2021 (Bundesverband der Arzneimittel-Hersteller e.V., 2022). We selected 1st place, cold remedies; 4th place, gastrointestinal tract remedies; and 10th place, antiallergics, from the list because these categories were sufficiently represented in both catalogues (Bundesverband der Arzneimittel-Hersteller e.V., 2022).

The 175 products from OP1 and the 109 products from OP2 represent the basic quantity used to present the top active ingredients. Other basic quantities were used for the guideline analysis.

### Comparison of active ingredients

Information from the respective catalogue was collected in tabular form. Each catalogue was analyzed and processed separately. The information from the catalogue on the product name; the pharmaceutical central number; the pharmaceutical form; the pack size; the prices and, if available, the active ingredient; the indication; and the age range were collected. The table was then supplemented with information from the corresponding package leaflets. For this purpose, the information that was missing in the catalogue was added to the table for some products in the previously documented categories.

The active ingredients category was then encoded in the tables for standardized data processing. For this purpose, each sub-category of the individual category was assigned a number and the meaning was documented, for example the number “1” for the active ingredient “X.” This resulted in encoding tables. This has the advantage that different formulations with the same meaning could be encoded in the same way and different formulations can therefore be standardized. The legends for the encoding can be found at the end of the tables.

The results were recorded in evaluation tables and presented graphically. Up to this point, the two catalogues have been considered separately. To compare the results, the analysis tables of both catalogues were merged, and a joint graphical evaluation was created.

### Analysis of conformity with guidelines

**Fig. 2:**
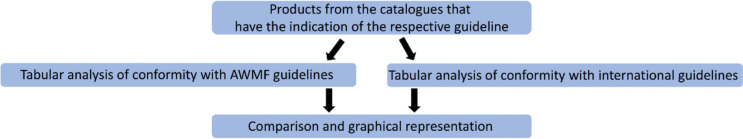
Graphical representation of the methods for comparing the guidelines with the product catalogues in a flow chart

To analyze the products offered in the catalogues, nine indications were selected for which the corresponding national guideline from the AWMF guideline register was used. That can be found in Table S1. In addition, international guidelines or national guidelines from Great Britain were selected for all nine indications, which can also be found in Table S1. For the sake of simplicity, the term “international guidelines” will be used in the following text to distinguish them from the German AWMF guidelines. The guidelines of the World Gastroenterology Organization (WGO) were used for diseases of the gastrointestinal tract, guidelines from the database of the National Institute of Health and Care Excellence (NICE) in Great Britain were used for cough and sore throat, and various European guidelines were used for urticaria, rhinosinusitis, and hemorrhoidal disease. Figure 2 shows the methodological procedure for the analysis of conformity with the respective guidelines of the online catalogues of OP1 and OP2.

In each case, the products from the catalogues which, according to the catalogue, had the indication of the respective guidelines were considered. The analysis was carried out in tabular form, indicating guideline agreement and the location of the recommendation in the guideline. It was checked whether the active ingredients of the products, the active ingredient group, the product name, or the product group were listed in the guideline and which assessment was represented by the guideline authors. After the results were collected and presented separately for national and international guidelines, the results were compared with each other.

The analysis of the nine guidelines only included some of the products considered in the catalogues due to the fact that not all indications are covered by the guidelines. Consequently, a total of 126 different products were included for OP1, which means 66.3% of the products analyzed in the areas of allergy, colds, and gastrointestinal tract from OP1 were included as well as 72 different products for OP2, which means 66.1% of the products analyzed from the OP2 catalogue. Some of the products were included in multiple guidelines if the products had multiple matching indications.

## Results and discussion

### Overview of product catalogues

**Fig. 3:**
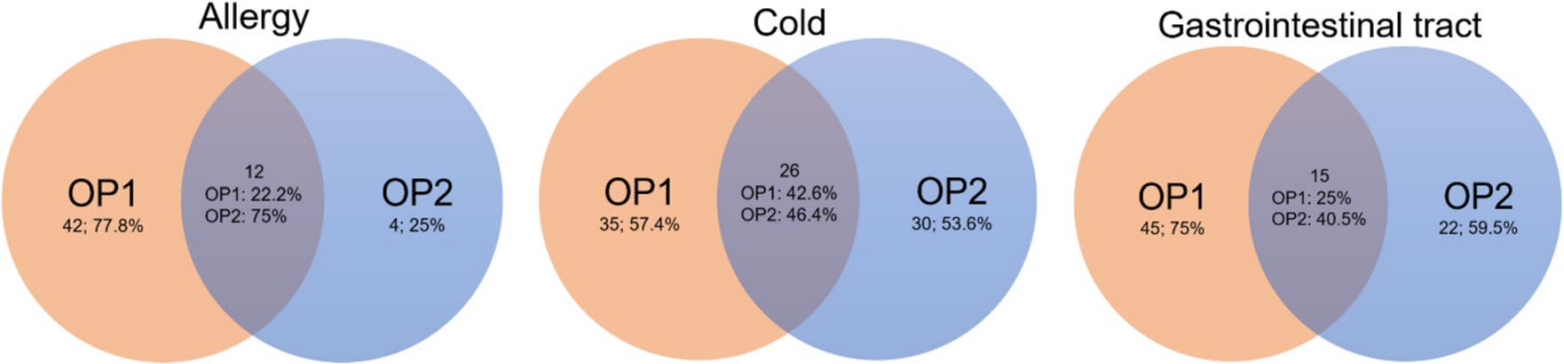
Illustration of the intersections (same products) of the products offered in the product catalogues of the online pharmacies OP1 and OP2 divided by indication category in three Venn diagrams; OP1 shown in orange and OP2 in blue

There are many self-medication products, which is why the range of products offered by the two online pharmacies differs in their catalogues. Figure 3 illustrates the proportion of products offered in both product catalogues, divided into three indication categories. Twenty-six of the products for colds appear in both catalogues, so this represents 42.6% of the cold products in OP1 and 46.4% in OP2. Both online pharmacies offer approximately the same number of products in the cold category (OP1 61; OP2 56), which is why the percentage of identical products is closest here. In the allergy category, 12 products appear in both catalogues, which accounts for 22.2% of all products in the allergy category for OP1 and 75% for OP2. This is where the percentage of identical products is furthest apart, as OP1 offers 54 products and OP2 only 16 products in its catalogue, meaning that OP2 has the maximum percentage of common products in the allergy area, whereas OP1 has the minimum. For the gastrointestinal tract indication area, 15 products are represented in both catalogues, which corresponds to 25% of the products in this category of OP1 and 40.5% of OP2.

### Active ingredients

**Fig. 4:**
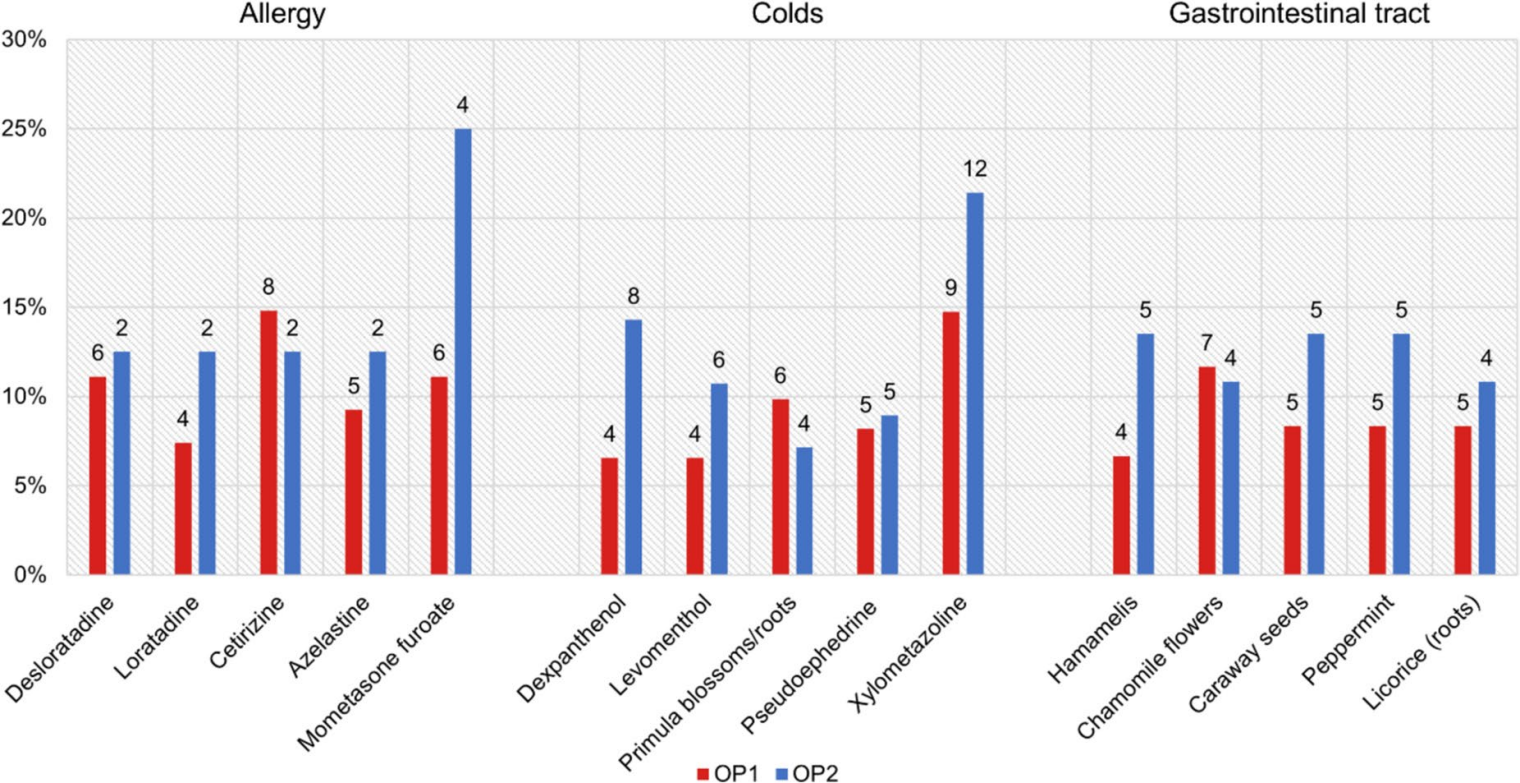
Top five active ingredients of the product catalogues of OP1 and OP2 in a bar chart. In blue OP2, in red OP1. The absolute figures represent the number of occurrences of the active ingredient in the products

Figure 4 shows the five most frequently occurring active ingredients for each of the three categories analyzed. To identify these five active ingredients, we calculated the sum of the occurrence for each active ingredient in both catalogues.

For the allergy category, all of the top five active ingredients are chemical-based. This is also reflected in the fact that only chemical drugs are offered in the OP2 catalogue and only two homeopathic drugs are offered in in OP1, which accounts for 3.7% of the OP1 range for the allergy category (see Fig. S1). In the allergy category, the active ingredients desloratadine, loratadine, cetirizine, and azelastine are found in 12.5% (2) of OP2’s products. In OP1, cetirizine is the most common active ingredient with 14.8% (8), while desloratadine is present in 11.1% (6) of the products in the OP1 catalogue. Azelastine accounts for 9.3% (5) and loratadine for 7.4% (4) of the OP1 products. These four active ingredients are 2nd generation H_1_ receptor antagonists and can be used for type 1 allergies (Freissmuth et al. 2020; Seifert 2018). They are characterized by a rapid onset of action with local application and can therefore be used well for allergic rhinitis or conjunctivitis (Seifert 2018). Loratadine, desloratadine, and cetirizine are only offered as tablets in the catalogues. Azelastine is offered both as a nasal spray and as eye drops.

By far, the most common active ingredient in the OP2 catalogue for the allergy category is mometasone furoate with 25% (4), which is represented as an active ingredient in 11.1% (6) of the products in OP1. This is a glucocorticoid receptor agonist that is used for local administration in allergic rhinitis (https://www.gelbe-liste.de/wirkstoffe/Mometason_40272; last accessed 10 April 2024). In the catalogues, mometasone furoate is only offered in the form of nasal sprays. The active ingredients mometasone furoate and desloratadine switched from prescription to pharmacy-only (non-prescription) status in 2016 and 2020, respectively (Bundesverband der Arzneimittel-Hersteller e.V., 2023).

In the cold category, two of the top five active ingredients, levomenthol and primrose flower/root extract, are of herbal origin. Looking at the active ingredients of all the drugs offered for the cold category, almost 25% of the products in OP1 are of herbal origin, and 8.2% of the products are homeopathic (see Fig. S1). In OP2, about 20% of the products are herbal and 3.4% are homeopathic (see Fig. S1). Most products in both catalogues are chemical drugs (see Fig. S1).

Xylometazoline is the most common active ingredient in both OP2 with 21.4% (12) and OP1 with 14.3% (9). This is an alpha_1_-adrenoreceptor agonist, which can be used locally with its vasoconstrictive effect in conjunctivitis or to reduce swelling in rhinitis (Seifert 2018). In the catalogues, xylometazoline is offered both as a monopreparation and in combination with dexpanthenol in the form of nasal sprays.

The active ingredient dexpanthenol is the second most common active ingredient in OP2 with 14.3% (8) and occurs in OP1 with 6.6% (4). It is the provitamin 5 and is applied topically, whereby it is converted to vitamin B5 in the body and has a healing-promoting effect (https://www.gelbe-liste.de/wirkstoffe/Dexpanthenol_195; last accessed 10 April 2024). For this reason, it is offered in cold preparations in the catalogues as a wound ointment as well as an additional active ingredient in decongestant nasal sprays.

Levomenthol is present in 10.7% (6) of the products of OP2 and in 6.6% (4) of the products of OP1. It is also called (−)-menthol and is contained in the essential oils of the plant genus *Mentha* (https://de.wikipedia.org/wiki/Menthol; last accessed 10 April 2024). It is postulated that (levo)-menthol exerts analgesic, local anesthetic and antimicrobial effects, which is why it is suitable for rhinitis, general colds, and inflammation of the throat (https://flexikon.doccheck.com/de/Menthol; last accessed 10 April 2024). In the catalogues, levomenthol is offered in combination preparations in the form of lozenges, cold ointment, nasal stick, or liquid.

Extracts of primrose flowers or roots are contained in 7.1% (4) of the products in the OP2 catalogue and 9.8% of the products in OP1, which makes it the second most common active ingredient. Primrose extracts are mainly used to loosen mucus in coughs due to their triterpene saponins, which are purported to exert a secretolytic and expectorant effect (https://www.apotheke-adhoc.de/rubriken/detail/erkaeltungs-tipps/primelwurzel-gelbes-hustenwunder-heilpflanzen-gegen-erkaeltung-erkaeltungstipp/; last accessed 10 April 2024). In the catalogues, the extracts of primrose are offered in the form of liquid or tablets in combination preparations.

Pseudoephedrine is contained in 8.9% (5) of the products in the OP2 catalogue and in 8.2% (5) of OP1. It is an indirect sympathomimetic and is found in combination preparations with analgesic active ingredients for colds (https://www.gelbe-liste.de/wirkstoffe/Pseudoephedrin_18255; last accessed 10 April 2024). Due to its vasoconstrictor effects, it can be used to reduce swelling of the mucous membranes (https://www.gelbe-liste.de/wirkstoffe/Pseudoephedrin_18255; last accessed 10 April 2024). In the catalogues, it is offered in the form of tablets in combination preparations with paracetamol, acetylsalicylic acid, or ibuprofen. Pseudoephedrine is a highly controversial active ingredient in the field of self-medication, mainly due to its adverse drug effects (Bade and Bendas 2023). In 2011, packs containing more than 720 mg of pseudoephedrine were re-switched to prescription status (Bundesverband der Arzneimittel-Hersteller e.V., 2023). The warning about severe skin reactions then had to be added as early as 2018 (Borsch 2018). Most recently in 2023, a safety review was launched by the European Medicines Agency (EMA), which now recommends the inclusion of new contraindications and warnings for products containing pseudoephedrine (Rößler 2023).

All active ingredients in the top five in the gastrointestinal tract area are of herbal origin. With 13.5% (5) each, the active ingredients witch hazel, caraway, and peppermint occur most frequently in the OP2 catalogue. The active ingredients chamomile flowers and licorice (roots) are each contained in 10.8% (4) of the products in the OP2 catalogue. In the OP1 catalogue, chamomile flowers are the most common active ingredient in the gastrointestinal tract area with 11.7% (7). The active ingredients caraway, peppermint, and licorice root each account for 8.3% (5) in OP1. Witch hazel is found in 6.7% (4) of the products in OP1. OP2 offers just as many herbal as chemical drugs, 46% each, while only 21.7% of the drugs offered in OP1 are herbal (see Fig. S1).

Witch hazel (*Hamamelis*) has supposedly anti-itching and anti-inflammatory properties, which is why it has established itself as a treatment for hemorrhoids (https://www.apotheken-umschau.de/medikamente/heilpflanzen/zaubernuss-pflanzliches-mittel-bei-hautproblemen-735813.html; last accessed 10 April 2024). The catalogues contain witch hazel in the form of tablets and ointments.

Chamomile flowers are assumed to have antibacterial, anti-inflammatory, and antispasmodic properties (https://www.medikamente-per-klick.de/apotheke/ernaehrungslexikon/kamille/; last accessed 10 April 2024). The essential oil of peppermint is said to have an antispasmodic, bile-flow-promoting, and flatulence-promoting function (Hierl 2019). Caraway supposedly has anti-inflammatory, antispasmodic, and flatulence-promoting properties (https://flexikon.doccheck.com/de/K%C3%BCmmel; last accessed 10 April 2024). The properties of licorice root are allegedly similar: antibacterial, antispasmodic, and anti-inflammatory (https://de.wikipedia.org/wiki/Echtes_S%C3%BC%C3%9Fholz; last accessed 10 April 2024). As these four active ingredients are very similar, they are used in combination preparations for functional gastrointestinal complaints, flatulence, or gastritis in the gastrointestinal tract. The catalogues contain these active ingredients in solutions, capsules, or tablets.

### AWMF guidelines

Nine guidelines from the AWMF guideline register were considered. The AWMF defines guidelines as statements that are developed based on the evaluation of evidence and risk-benefit assessment (https://www.awmf.org/leitlinien; last accessed 23 July 2024). The guidelines contain recommendations for action that have been formulated on the basis of the extent state of knowledge (https://www.awmf.org/leitlinien; last accessed 23 July 2024). The AWMF guidelines are classified into three groups: S1 (expert groups justify recommendations for action), S2 (evidence- or consensus-based guidelines), and S3 (evidence- and consensus-based guidelines) (Nothacker et al. 2014). The aim of the guidelines is to ensure an adequate quality of care by providing medical and other professionals with guidance on the evidence-based approach to the treatment of specific diseases (https://www.awmf.org/leitlinien; last accessed 23 July 2024). Guideline recommendations should not be understood as mandatory, but it is always necessary to check the applicability to the individual patient context and to adapt the guideline recommendation if necessary to offer the patient the best possible care (https://www.awmf.org/leitlinien; last accessed 23 July 2024).

Only 126 products of all OP1 products (66.3%) and 72 products of all OP2 products (66.1%) were included in the guideline analysis of the product catalogues, as the other products had indications that were not covered in any of the guidelines considered. In each case, it was checked whether the drug name, the active ingredient, or the group of active ingredients was mentioned in the guideline.

**Fig. 5:**
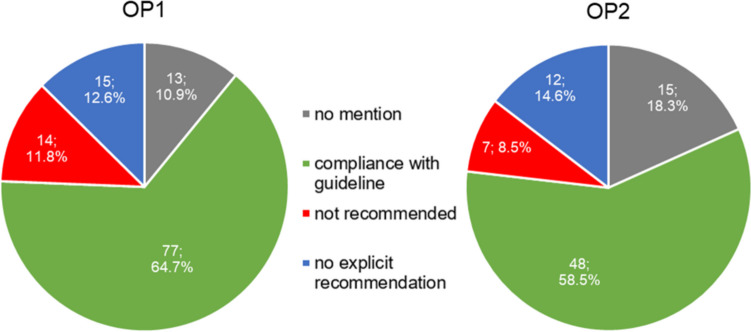
Illustration of the analysis of the conformity of the guidelines from the AWMF register with the products from the product catalogues of OP1 (left) and OP2 (right) in two pie charts

Figure 5 shows the conformity of the AWMF guidelines with the products offered from the OP1 and OP2 catalogues. The treatment recommendation was complied with by 65.5% (78) of the products from OP1 and 61.1% (50) of the products from OP2.

**Fig. 6:**
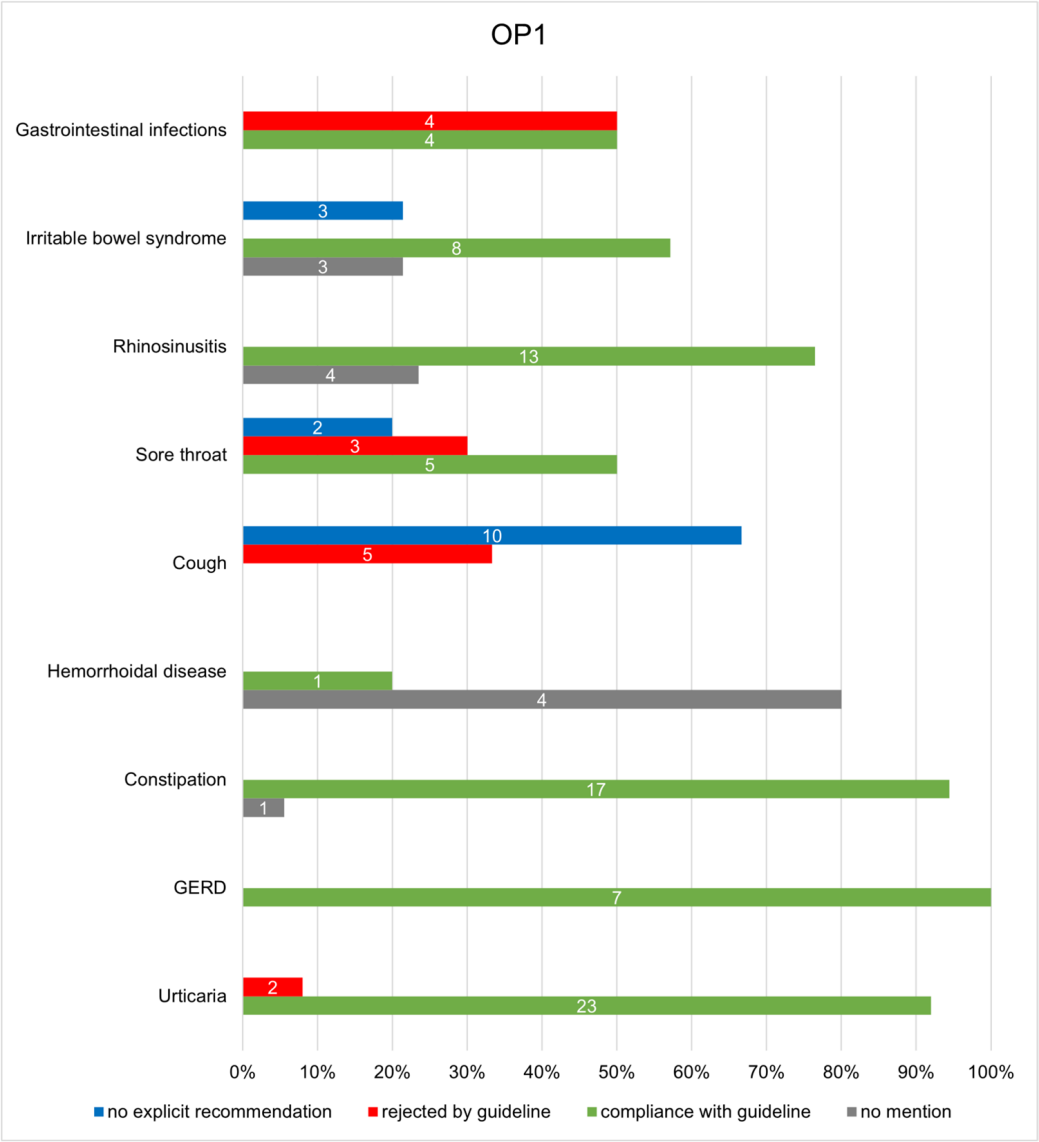
Illustration of the analysis of the conformity of the individual guidelines with the advertised products of OP1 in a bar chart. The absolute figures represent the number of products for the respective category

**Fig. 7:**
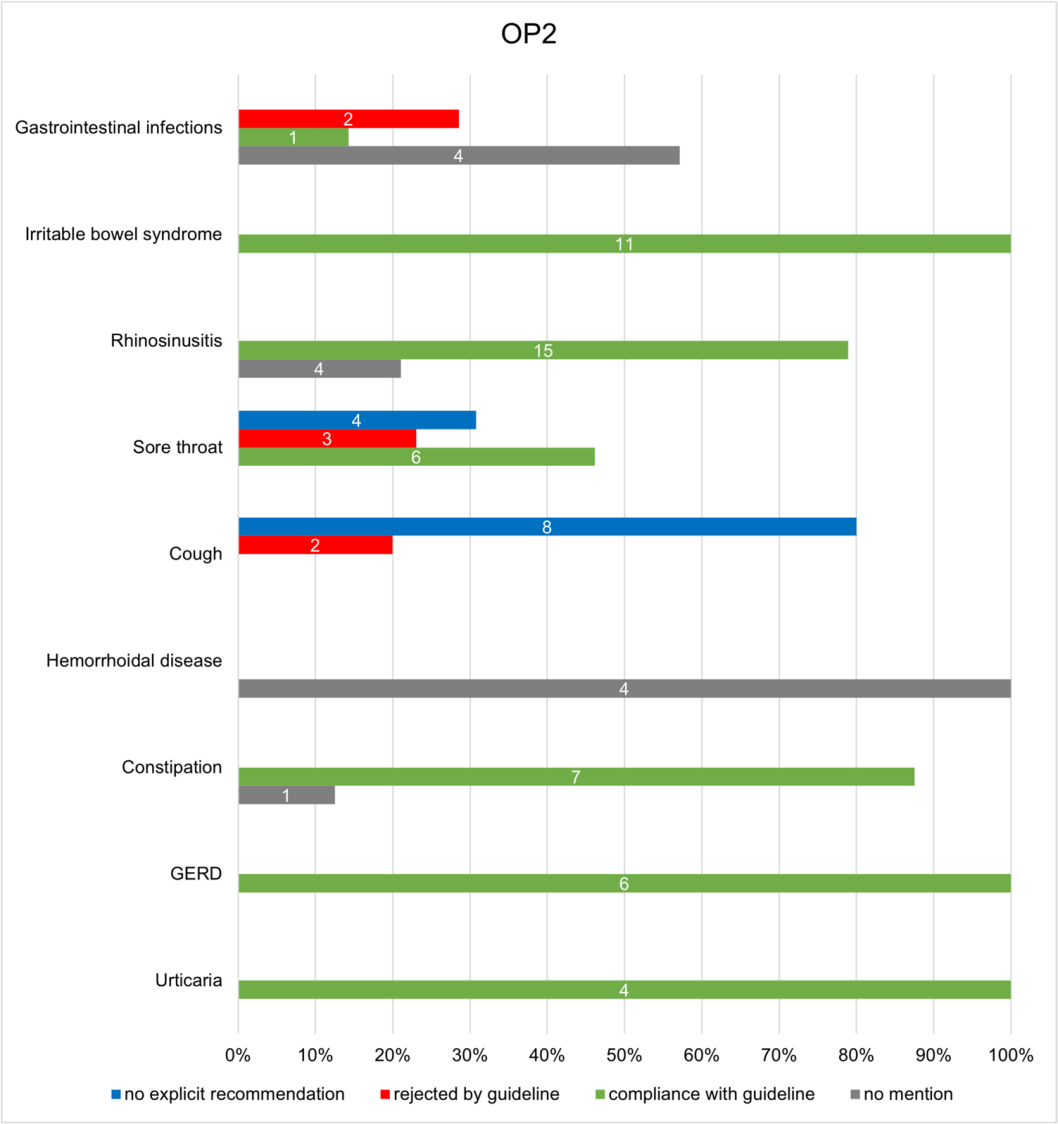
Illustration of the analysis of the conformity of the individual guidelines with the advertised products of OP2 in a bar chart. The absolute figures represent the number of products for the respective category

Figures 6 and 7 show the conformity of the individual guidelines with the advertised products in the OP1 and OP2 catalogues. Looking at the individual indications, it is positive to note that in both catalogues, all products offered for gastroesophageal reflux disease (GERD) are compliant with the guidelines, which means recommended. In addition, 92% of OP1 and 100% of OP2 products offered for urticaria, 94.4% of OP1 products and 87.5% of OP2 products for constipation, and 100% of OP2 products for irritable bowel syndrome are recommended by the respective guideline.

Some of the advertised preparations from the catalogues are not mentioned in the guidelines either as a drug name, as an active ingredient, or as an active ingredient group, or not in the present combination, which is why no evaluation could be carried out for them. This includes 10.1% (12) of the products of OP1 and 15.9% (13) of the products of OP2. As no recommendation was therefore made for these products in the guidelines, it can be assumed that these products are not the treatment of first choice. This mainly concerns products for the indication of hemorrhoidal disease, as 80% (4) of the products of OP1 and 100% (4) of the products of OP2 are not mentioned in the guideline. These are products with witch hazel leaf and twig extract, which were offered in both catalogues in the form of suppositories and ointments but were not covered in the associated guideline. In addition, 57.1% (4) of the products offered by OP2 for gastrointestinal infections were also not mentioned in the guideline. This concerns products with Indian psyllium husk as the active ingredient.

Furthermore, 12.6% (15) of the products analyzed for conformity with guidelines in OP1 and 14.6% (12) in OP2 were not explicitly recommended by the guidelines. For the indication of cough, this includes phytotherapeutics, for example mono- or combination preparations with the active ingredients ivy, thyme, primrose root extract, or pelargonium root extract (Krüger et al. 2021). This applies to 66.7% (10) of the products for cough from OP1 and 80% (8) from OP2 (see Fig. S4, S5). The guideline states that no sufficient clinical effects could be determined, which is why no explicit recommendation was made for these phytotherapeutics in the guideline (Krüger et al. 2021). Instead, the guideline allows the use of phytotherapeutics as an additional option if the patient wishes to use them (Krüger et al. 2021).

The guidelines did not recommend 11.8% (14) of OP1 products and 8.5% (7) of OP2 products. Table 1 presents a summary of the studies referenced in the guidelines for products from the catalogues that have been identified as non-compliant with the guidelines. Non-compliance affects 33.3% (5) of the products in OP1 and 20% (2) of the products in OP2 for the indication of cough. In both catalogues, these are expectorants, for example with the active ingredients acetylcysteine or ambroxol, which are not recommended by the guidelines for acute cough, as no sufficient evidence of efficacy could be established (Krüger et al. 2021). Regarding this issue, the guideline refers to four studies, two of which, however, relate exclusively to cough in the context of chronic bronchitis and are therefore not decisive for the treatment of acute cough. The two studies on an acute cough that were listed in the guideline are a randomized controlled trial (RCT) by Matthys et al. from 2000, which advocates the use of ambroxol, and a systemic review by Smith et al. from 2014, which did not find any convincing results on the efficacy of expectorants, so that the authors of the guideline justified the negative recommendation (Krüger et al. 2021).

**Table 1:**
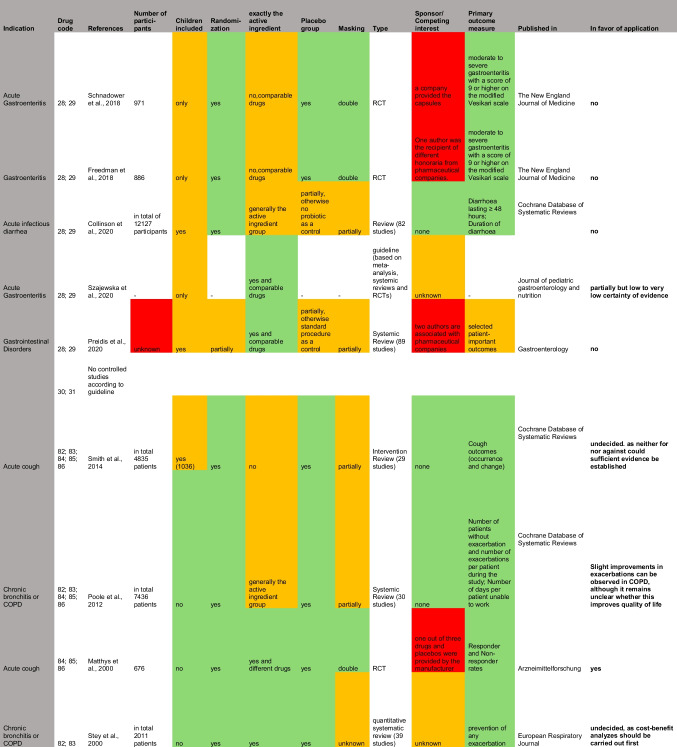

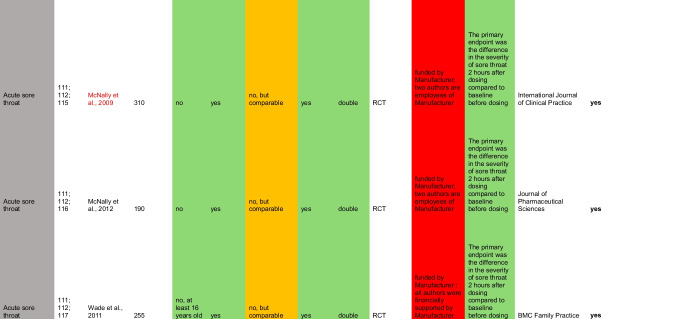
Detailed analysis of the studies referenced to justify the negative recommendations in the AWMF guidelines, presented in a table. Green parameters have a high scientific value. Red parameters reduce the scientific value. Orange parameters neither increase nor decrease the scientific value of the study

In the case of gastrointestinal infections, the associated guideline strongly advises against the use of probiotics and certain antidiarrheals, for example with the active ingredients charcoal or myrrh (Addo et al. 2023), which accounts for 50% (4) of the products considered in OP1 and 28.6% (2) in OP2. All five listed references on the efficacy of probiotics were unable to establish convincing efficacy, so that the authors of the guideline expressed opposition to the use of probiotics in the treatment of acute gastroenteritis (Addo et al. 2023).

For the indication of sore throat, three products with local antiseptics are offered in both catalogues, which are not recommended by the guidelines (Oltrogge et al. 2020). This accounts for 30% of products for the indication of sore throat in OP1 and 23.1% in OP2. Although the three studies cited in the guideline are all in favor of the use of antiseptic lozenges, it should be noted that the studies are all supported by the manufacturer, which is why bias and competing interests cannot be ruled out.

Looking at the results, from a consumer’s perspective, it must be criticized that around 10% of the products advertised by both online pharmacies received a negative recommendation and should therefore not be used. As the guidelines were primarily written for healthcare professionals, it is impossible for laypersons to understand which products are effective and useful. Therefore, on the one hand, educational campaigns should be offered to consumers, for example to explain the myth of the effectiveness of expectorants. On the other hand, pharmacies should not actively promote products that have no evidence of efficacy. But of course, pharmacies will have a conflict of interest here, since guideline-directed recommendations would likely reduce sales. Thus, the best solution would be to remove ineffective products from the market. However, this will be most likely counteracted by lobbyism, as the sale of expectorants, for example, is a lucrative market. In 2022, sales of cough medicines in Germany amounted to 522 million euros, making them the third highest-selling indication group (Bundesverband der Arzneimittel-Hersteller e.V. 2023). Accordingly, this guideline analysis underpins the problem that pharmacies also offer many products whose evidence is unconvincing or non-existent, making it impossible for laypersons to differentiate which product on offer is effective.

### International guidelines

Looking beyond the national context, there are many other guidelines from various societies and institutions. Guidelines from the National Institute for Health and Care Excellence (NICE) in Great Britain and the World Gastroenterology Organization (WGO) were used for comparison. Table S1 shows which guideline was used for which indication.

**Fig. 8:**
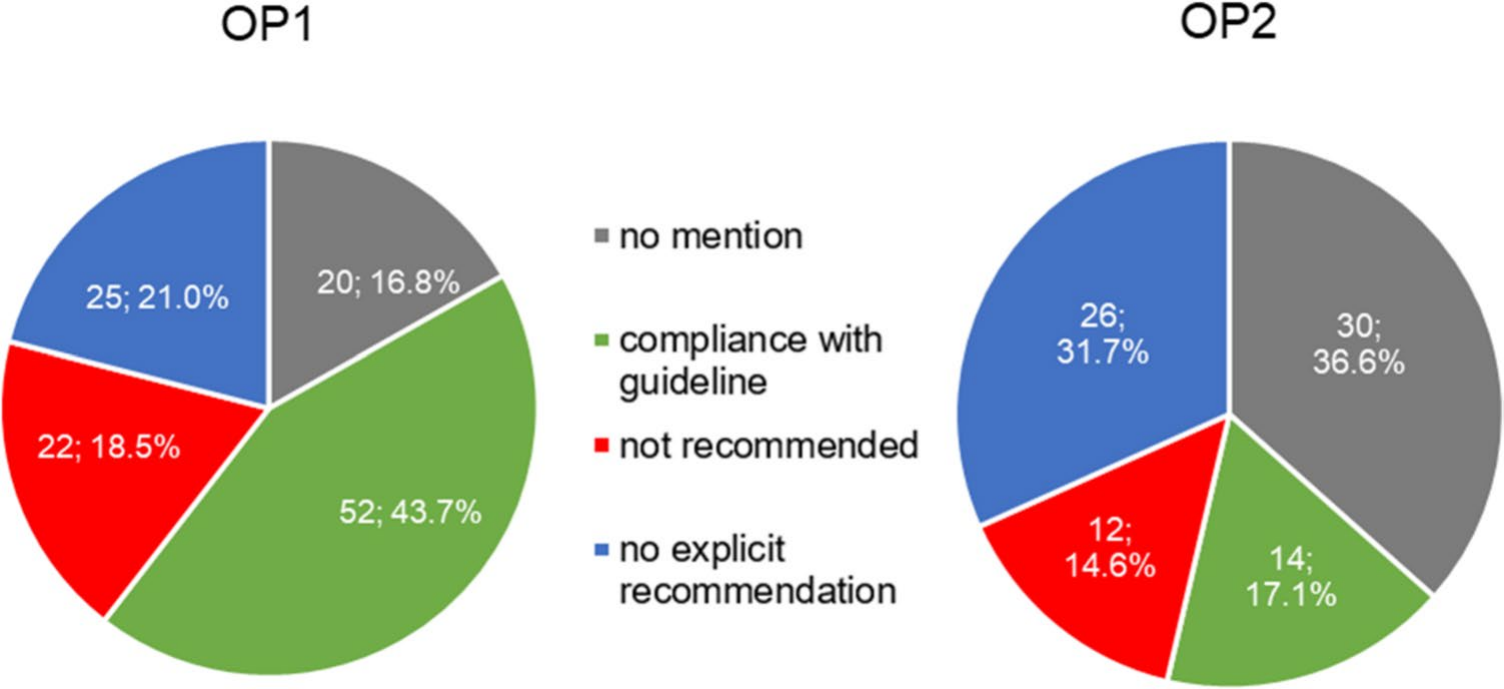
Illustration of the analysis of the conformity of international guidelines with the products from the product catalogues of OP1 (left) and OP2 (right) in two pie charts

Figure 8 shows the conformity of the international guidelines with the products offered from the OP1 and OP2 catalogues. The treatment recommendation was complied with by 43.7% (52) of the products from OP1 and 17.1% (14) from OP2. Table 2 contains a comparison of the percentage assessment of the national AWMF and international guidelines.

**Table 2:**
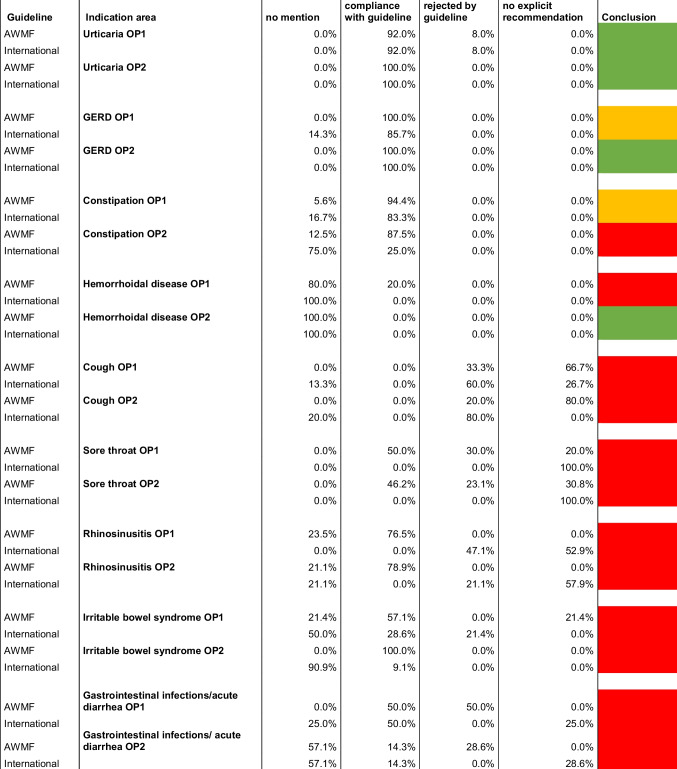
Overview of the compliance of AWMF and international guidelines with the catalogues of OP1 and OP2; the last column compares the results between AWMF and international guidelines for an indication within a catalogue; in green complete agreement, in yellow deviations in the categories up to 15%, in red more than 15% deviations

In the OP1 catalogue, the products for the indication urticaria performed best, as 92% of the products offered complied with the guideline recommendations. In the OP2 catalogue, all products offered for the indications of urticaria and GERD were recommended by the respective international guidelines.

A total of 16.8% (20) of the advertised preparations of OP1 and 36.6% (30) of OP2 are not referenced in the guidelines, either as a drug name, as an active ingredient, as an active ingredient group, or as part of the current combination. This is why an evaluation could not be conducted in this case. Due to the lack of mention, it can be concluded that these products should not be used as first-line therapy. This applies to 100% of the advertised products for hemorrhoidal disease in both catalogues. Also, 50% of the products offered by OP1 and 90.9% by OP2 for the indication of irritable bowel syndrome are not mentioned in the associated guidelines. In addition, 25% (OP1) and 57.1% (OP2) of the products for acute diarrhea and 75% of the products from the OP2 catalogue for constipation are not mentioned in the guidelines.

Products that were not advised against, but for which no explicit positive recommendation was given, account for 21% (25) in OP1 and 31.7% (26) in OP2. This applies to 100% of the products for the indication of sore throat and over 50% of the products for rhinosinusitis in both catalogues. Also, 25% (OP1) and 28.6% (OP2) of the products offered for acute diarrhea were not explicitly recommended.

Negative recommendations were given to 18.5% (22) of the products from the OP1 catalogue and 14.6% (12) of the products from the OP2 catalogue in comparison with the international guidelines. For the OP1 catalogue, 60% of the products for cough, 47.1% of the products for rhinosinusitis, 21.4% of the products for functional gastrointestinal complaints, and 8% of the products for urticaria were recommended against by the guidelines. In OP2, the guidelines advised against 80% of products for acute cough and 21.1% of products for rhinosinusitis. In the area of cough, the authors of the guideline expressed opposition to the use of mucolytics and herbal active ingredients like primrose, ivy, and thyme (National Institute for Health and Care Excellence, 2019). The guideline on rhinosinusitis advises against herbal products (Orlandi et al. 2021). According to the guidelines on irritable bowel syndrome, there is no evidence for the use of products containing the active ingredient simethicone (Quigley et al. 2015). The guideline on the management of urticaria states that the use of 1st generation H_1_-receptor antagonists is not recommended due to strong evidence regarding adverse effects (Zuberbier et al. 2022).

### Comparison of guidelines

A comparison of the results of the German (AWMF) and international guidelines reveals heterogeneity (see Figs. 5 and 8). The AWMF guidelines comparison with the international guidelines shows an increase in products that are not mentioned in the guideline (see Figs. 5 and 8). While there was an increase of +5.9% for OP1 in the international guidelines compared to the AWMF guidelines, the number of products from the OP2 catalogue not mentioned in the guideline doubled from 18.3 to 36.6% (see Figs. 5 and 8). This is particularly true for the guideline on constipation, where 12.5% of the products in the OP2 catalogue were not mentioned in the comparison with the AWMF guideline, whereas this applied to 75% of the products in the comparison with the international guideline (see Table 2). The reason for this is that OP2 offers five products with the active ingredient psyllium, which was still included in the AWMF guideline but is not mentioned in the international guideline. The comparison of guidelines on irritable bowel syndrome also shows a significant increase in products that were not mentioned in the international guideline compared to the AWMF guideline (see Table 2). There is an increase in OP1 from 21.4% (AWMF) to 50% (international) and in OP2 from 0% (AWMF) to 90.9% (international) (see Table 2). The reason for this is that the international guideline only addresses Chinese herbal medicine, while other herbal active ingredients and combinations of active ingredients were not addressed. Regarding the OP1 catalogue, an increase in products not mentioned from 0% to a quarter of the products considered for the gastrointestinal infections/acute diarrhea guideline should also be mentioned, as coffee charcoal and herbal active ingredients were not addressed in the international guideline (see Table 2).

A comparison of the “no explicit recommendation” category shows that there is an increase in products from the AWMF guidelines to the international guidelines (see Figs. 5 and 8). The AWMF analysis showed 12.6% for the OP1 catalogue and 14.6% for the OP2 catalogue, whereas the comparison with the international guidelines showed 21% for OP1 and 31.7% for OP2 (see Figs. 5 and 8). The guideline results for the indications of sore throat, rhinosinusitis, and gastrointestinal infections/acute diarrhea are particularly noteworthy, with an increase in in the number of products that did not receive an explicit recommendation from the associated international guideline (see Table 2). The international guideline on sore throat classified 100% of the products from both catalogues in this category, as the guideline argues that the use of both medicinal and non-medicinal lozenges and throat sprays is possible in the context of self-medication, but that no significant improvement is to be expected (National Institute for Health and Care Excellence 2018). In the AWMF guideline, herbal products were not explicitly recommended, as the guideline only advocates their use if requested by the patient (Oltrogge et al. 2020). When comparing the guidelines on rhinosinusitis, the international guideline also shows a sharp increase in products that did not receive an explicit recommendation to over 50% (see Table 2). This concerns products with the active ingredient xylometazoline, which was offered several times in both catalogues as a topical decongestant in the form of nasal sprays. The guideline argues that risks and adverse effects must be considered and that there is no convincing evidence for its effectiveness (Orlandi et al. 2021). In the AWMF guideline, decongestants were recommended if they are not used for more than 10 days (Stuck et al. 2017).

There was less conformity in both catalogues when compared with the international guidelines than with the AWMF guidelines (see Figs. 5 and 8). All other three categories showed an increase at the expense of conformity. In comparison with the AWMF guidelines, 65.5% (OP1) and 61% (OP2) of the products received a recommendation, whereas in the analysis with international guidelines 43.7% (OP1) and 17.1% (OP2) of the products were recommended (see Figs. 5 and 8). The guidelines on rhinosinusitis, sore throat, constipation, and irritable bowel syndrome showed strong differences in conformity (see Table 2). In the guideline on rhinosinusitis, the AWMF recommended decongestants such as xylometazoline, which was not explicitly recommended in the international guideline (Orlandi et al. 2021; Stuck et al. 2017). In the AWMF guideline on a sore throat, non-medicated and medicated lozenges with local anesthetics received a recommendation, while the international guideline does not explicitly recommend them (National Institute for Health and Care Excellence 2018; Oltrogge et al. 2020). In the guideline on constipation and irritable bowel syndrome, the differences are due to the lack of mention of psyllium and/or the lack of mention of herbal active ingredients in the international guideline.

The proportion of drugs that were not recommended in the guideline increased from 11.8% (OP1) and 8.5% (OP2) in the AWMF guidelines to 18.5% (OP1) and 14.6% (OP2) in the international guidelines (see Figs. 5 and 8). Guidelines that showed large differences in comparison included the indications of cough, rhinosinusitis, and irritable bowel syndrome (see Table 2). In both guidelines on cough, expectorants were not recommended, but the international guideline additionally advised against herbal products, for example with the active ingredients thyme or ivy, which is the reason for the increase in products that were advised against by the international guideline (Krüger et al. 2021; National Institute for Health and Care Excellence, 2019). In the AWMF guidelines on rhinosinusitis and irritable bowel syndrome, no negative recommendation was made for any product in the guideline analysis, whereas this was the case in the analysis of the international guideline for both indications (see Table 2). The reason for this is that herbal active ingredients are not recommended in the international guidelines on rhinosinusitis (Orlandi et al. 2021). The international guideline on irritable bowel syndrome advised against simeticone, which was not explicitly recommended in the AWMF guideline (Layer et al. 2021; Quigley et al. 2015).

When interpretating the different results regarding the conformity of the catalogues of OP1 and OP2 with the AWMF and international guidelines, it should be noted that the AWMF guidelines refer specifically to active ingredients approved in Germany and that the organization and status of the German healthcare system were explicitly considered in the analysis. The international guidelines are either based on a different healthcare system (NICE guidelines from Great Britain) or published by the WGO, which makes global recommendations in its guideline and is therefore not adapted to the availability of individual active ingredients in specific countries and the possibilities of national healthcare systems.

**Fig. 9:**
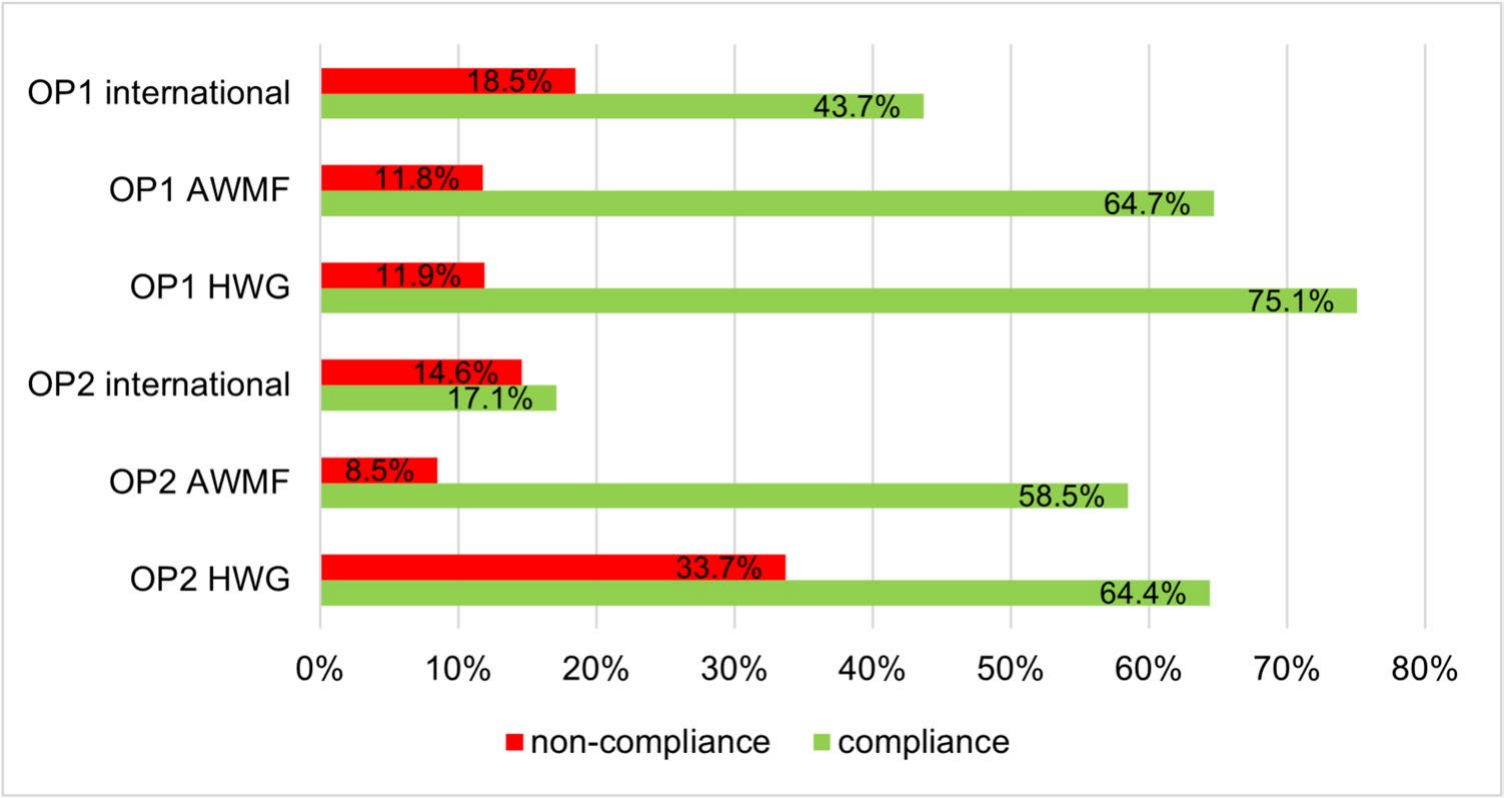
Comparison of the (non-)conformity of the product catalogues of OP1 (top) and OP2 (bottom) with the HWG (according to Barlage and Seifert 2024) and the guidelines in a bar chart

In a recent paper analyzing the catalogues of online pharmacies, serious non-conformity with the Therapeutic Products Advertising Act (Heilmittelwerbegesetz; HWG) was noted (Barlage and Seifert 2024). Only 75.1% of OP1 and 64.4% of OP2 comply with the law (Barlage and Seifert 2024). Figure 9 illustrates the conformity or non-conformity of the product catalogues with the HWG and the guidelines. Overall, the extent of non-conformity with respect to the HWG and guidelines is similar. These deficiencies can have substantial consequences for consumer safety. Consumers buy products advertised by online pharmacies in their catalogues because they assume that it is a product that is effective for the advertised indication. However, a lack of mandatory information according to the HWG (Barlage and Seifert 2024) and a lack of evidence, as listed in the guidelines, can lead to consumers buying a product that does not provide the best possible relief for their current complaints. In the future, rules and regulations should be established to better protect consumers when buying drugs.

## Limitations of the analysis

This analysis is based exclusively on free and public sources. The analysis only included products from three categories of the catalogues, so that only certain parts of the catalogues were considered and the results do not represent the entire catalogue. In addition, the products were selected based on the titles of the categories in the catalogues, so it is not possible to exclude the possibility that other products belonging to the allergy, cold, or gastrointestinal tract categories were listed under different titles in the catalogues and were therefore not included in this analysis.

The analysis of conformity with the guidelines was limited to a subset of all products considered from the catalogues of the online pharmacies, as only nine selected guidelines and their associated indications were included. In addition, the S2k guideline on rhinosinusitis is from April 2017 and thus bears the AWMF’s note that it has not been updated for more than 5 years, which is why the recommendations are only up to date to a limited extent. The international guidelines are heterogeneous, as there is no institution that publishes uniform guidelines for all indications, meaning that differences arise due to different working methods in the guidelines. It should also be noted that the AWMF guidelines are adjusted to the German healthcare system, whereas the international guidelines are not. The AWMF guidelines included fewer products in the “not mentioned” category, as the guideline only analyzed nationally approved products. In contrast to the German guidelines, the international guidelines also included products that are not approved in Germany, resulting in a larger product pool being considered. It is therefore imperative that any comparison of the products listed in the catalogues of German online pharmacies with the international guidelines be conducted with caution.

## Conclusions

We observed considerable deviations of the catalogues of online pharmacies with established AWMF guidelines. The analysis of products from OP1 and OP2 revealed that only 65.5% (78) and 61.1% (50) respectively conformed to the AWMF treatment recommendations. In addition, there are even greater deviations from the international guidelines, where only 43.7% of OP1 products and 17.1% of OP2 complied with the recommendations. The greatest non-conformity with the AWMF guidelines is for products for gastrointestinal infections and with the international guidelines for cough products. Thus, substantial portions of the catalogues are not evidence-driven but rather market-driven. It can be assumed that large portions of the self-medication market have no solid scientific basis. From a scientific perspective and for reasons of consumer protection, non-evidence-based self-medication should be stopped, not just because of the lack of efficacy but also because self-medication could cause serious adverse reactions and interactions with doctor-prescribed drugs. A feasible starting point for future studies on this topic would be to systematically assess intoxications caused by self-medications without scientific evidence for effectiveness.

Future revisions of AWMF guidelines should consider international guidelines to a greater extent to obtain a broader scientific conformity. Regarding urticaria and gastroesophageal reflux, international consensus is actually very good, but with regard to all other indication areas studied here, including highly market-relevant rhinosinusitis and irritable bowel syndrome, consensus of AWMF with international guidelines is poor. This is an indication that cultural and national idiosyncrasies may play a greater role in the AWMF guidelines than is generally appreciated. The best approach to resolve these discrepancies would be to conduct high-quality prospective randomized clinical trials. Furthermore, the establishment of an international organization responsible for the issuance of uniform guidelines for all indications would be highly beneficial.

For the future, it would be desirable to check whether the advice given by pharmaceutical professionals in brick-and-mortar pharmacies is in accordance with the drugs recommended in guidelines for the specific indication or whether profit motives influence the advice and thus drugs that are advised against in the guidelines are increasingly recommended in pharmacies.

Overall, our analysis of the compliance of the HWG and our analysis of the compliance of guidelines have demonstrated that online pharmacy product catalogues are not a reliable information source for consumers, as their information content has too many shortcomings. The catalogues are more market-oriented than science-oriented. These deficiencies underline the necessity of a stricter surveillance of the HWG in the interest of consumer safety.

## Supplementary Information

Below is the link to the electronic supplementary material.

Supplementary file1 (DOCX 81 KB)

## Data Availability

All source data for this work are available upon reasonable request.

## Abbreviations

AWMF: Arbeitsgemeinschaft der Wissenschaftlichen Medizinischen Fachgesellschaften e.V (Association of the Scientific Medical Societies in Germany)
EMA: European Medicines Agency
GERD: Gastroesophageal reflux disease
HWG: Heilmittelwerbegesetz; Therapeutic Products Advertising Act
NICE: National Institute of Health and Care Excellence
OP: Online pharmacy
OTC: Over-the-counter
RCT: Randomized controlled trial
WGO: World Gastroenterology Organization

## References

[CR1] ABDA (2023) Die Apotheke: Zahlen Daten Fakten 2023: Statistisches Jahrbuch der ABDA. (ABDA – Bundesvereinigung Deutscher Apothekerverbände e. V.)

[CR2] Addo MM, Lohse AW, Stallmach A, Manthey CF, Epple H-J, Keller K-M, Lübbert C, Posovszky C, Ramharter M, Reuken P, Suerbaum S, Vehreschild M, Weinke T, Deutsche Gesellschaft für Gastroenterologie, Verdauungs- und Stoffwechselkrankheiten e.V. (2023) S2k-Leitlinie Gastrointestinale Infektionen

[CR3] Bade L, Bendas G (2023) Pseudoephedrin: Repetitorium. 2023

[CR4] Barlage L, Seifert R (2024) Insufficient compliance of the German Therapeutic Products Advertising Act in product catalogues of online pharmacies. Naunyn-Schmiedeberg’s Arch Pharmacol. 10.1007/s00210-024-03370-7 10.1007/s00210-024-03370-7 PMC11825602 39207596

[CR5] Borsch J (2018) Schwere Hautreaktionen durch Pseudoephedrin - Änderung der Produktinformation notwendig. PTAheute

[CR6] Bundesverband der Arzneimittel-Hersteller e.V. (2022) Der Arzneimittelmarkt in Deutschland: Zahlen & Fakten aus 2021

[CR7] Bundesverband der Arzneimittel-Hersteller e.V. (2023) Der Arzneimittelmarkt in Deutschland: Zahlen & Fakten aus 2022

[CR8] Collinson S, Deans A, Padua-Zamora A, Gregorio GV, Li C, Dans LF, Allen SJ (2020) Probiotics for treating acute infectious diarrhoea. Cochrane Database Syst Rev 12:003048. 10.1002/14651858.CD003048.pub4 10.1002/14651858.CD003048.pub4 PMC8166250 33295643

[CR9] Freedman SB, Williamson-Urquhart S, Farion KJ, Gouin S, Willan AR, Poonai N, Hurley K, Sherman PM, Finkelstein Y, Lee BE, Pang X-L, Chui L, Schnadower D, Xie J, Gorelick M, Schuh S (2018) Multicenter trial of a combination probiotic for children with gastroenteritis. N Engl J Med 379:2015–2026. 10.1056/NEJMoa1802597 30462939 10.1056/NEJMoa1802597

[CR10] Freissmuth M, Offermanns S, Böhm S (2020) Pharmakologie und Toxikologie: Von den molekularen Grundlagen zur Pharmakotherapie. 3., überarbeitete Auflage. (Berlin; Heidelberg: Springer)

[CR11] Hierl E-M (2019) Pfefferminze. (Stuttgart). https://www.deutsche-apotheker-zeitung.de/news/artikel/2019/11/21/pfefferminze

[CR12] Krüger K, Gehrke-Beck S, Holzinger F, Heintze C, Deutsche Gesellschaft für Allgemeinmedizin und Familienmedizin e.V. (2021) S3-Leitlinie Akuter und chronischer Husten. (Berlin)

[CR13] Layer P, Andresen V, Allescher H, Bischoff SC, Claßen M, Elsenbruch S, Freitag M, Frieling T, Gebhard M, Goebel-Stengel M, Häuser W, Holtmann G, Keller J, Kreis ME, Kruis W, Langhorst J, Lynen Jansen P, Madisch A, Mönnikes H, Müller-Lissner S, Niesler B, Pehl C, Pohl D, Röhrig-Herzog G, Schemann M et al (2021) Update S3-Leitlinie Reizdarmsyndrom: Definition, Pathophysiologie, Diagnostik und Therapie. Gemeinsame Leitlinie der Deutschen Gesellschaft für Gastroenterologie, Verdauungs- und Stoffwechselkrankheiten (DGVS) und der Deutschen Gesellschaft für Neurogastroenterologie und Motilität (DGNM). (Deutschen Gesellschaft für Gastroenterologie, Verdauungs- und Stoffwechselkrankheiten (DGVS); Deutschen Gesellschaft für Neurogastroenterologie und Motilität (DGNM))

[CR14] Lehmkuhl D, Meissner W, Neugebauer EAM (2011) Evaluation der “Initiative Schmerzfreie Klinik” zur Qualitätsverbesserung in der postoperativen Schmerztherapie. Eine Prospektive Kontrollierte Studie. Schmerz 25:508–515. 10.1007/s00482-011-1054-z 21786029 10.1007/s00482-011-1054-z

[CR15] Matthys H, Mey C, Carls C, Ryś A, Geib A, Wittig T (2000) Efficacy and tolerability of myrtol standardized in acute bronchitis. A multi-centre, randomised, double-blind, placebo-controlled parallel group clinical trial vs. cefuroxime and ambroxol. Arzneimittelforschung 50:700–711. 10.1055/s-0031-1300276 10994153 10.1055/s-0031-1300276

[CR16] McNally D, Simpson M, Morris C, Shephard A, Goulder M (2009) Rapid relief of acute sore throat with AMC/DCBA throat lozenges: randomised controlled trial: relief of acute sore throat with AMC/DCBA throat lozenges. Int J Clin Pract 64:194–207. 10.1111/j.1742-1241.2009.02230.x 19849767 10.1111/j.1742-1241.2009.02230.x PMC7202229

[CR17] McNally D, Shephard A, Field E (2012) Randomised, double-blind, placebo-controlled study of a single dose of an amylmetacresol/2,4-dichlorobenzyl alcohol plus lidocaine lozenge or a hexylresorcinol lozenge for the treatment of acute sore throat due to upper respiratory tract infection. J Pharm Pharm Sci 15:281–294. 10.18433/j31309 22579007 10.18433/j31309

[CR18] National Institute for Health and Care Excellence (2018) Sore throat (acute): antimicrobial prescribing. (National Institute for Health and Care Excellence)

[CR19] National Institute for Health and Care Excellence (2019) Cough (acute): antimicrobial prescribing. (National Institute for Health and Care Excellence)

[CR20] Nothacker M, Muche-Borowski C, Kopp IB (2014) 20 Jahre ärztliche Leitlinien in Deutschland - was haben sie bewirkt? Z Evid Fortbild Qual Gesundhwes 108:550–559. 10.1016/j.zefq.2014.10.012 25499107 10.1016/j.zefq.2014.10.012

[CR21] Ollenschläger G, Thomeczek C, Kirchner H, Oesingmann U, Kolkmann FW (2000) Leitlinien und Evidenz-basierte Medizin in Deutschland. Z Gerontol Geriatr 33:82–89. 10.1007/s003910050161 10851705 10.1007/s003910050161

[CR22] Oltrogge JH, Chenot JF, Schmiemann G, Weckmann G, Toepfner N, Berner R, Bickel M, Laskawi R, Windfuhr J, Krüger K, Deutsche Gesellschaft für Allgemeinmedizin und Familienmedizin e.V. (2020) S3-Leitlinie Halsschmerzen. (Berlin)

[CR23] Orlandi RR, Kingdom TT, Smith TL, Bleier B, DeConde A, Luong AU, Poetker DM, Soler Z, Welch KC, Wise SK, Adappa N, Alt JA, Anselmo-Lima WT, Bachert C, Baroody FM, Batra PS, Bernal-Sprekelsen M, Beswick D, Bhattacharyya N, Chandra RK, Chang EH, Chiu A, Chowdhury N, Citardi MJ, Cohen NA et al (2021) International consensus statement on allergy and rhinology: rhinosinusitis 2021. Int Forum Allergy Rhinol 11:213–739. 10.1002/alr.22741 33236525 10.1002/alr.22741

[CR24] Poole P, Black PN, Cates CJ (2012) Mucolytic agents for chronic bronchitis or chronic obstructive pulmonary disease. Cochrane Database Syst Rev (8):CD001287. 10.1002/14651858.CD001287.pub4 10.1002/14651858.CD001287.pub4 22895919

[CR25] Preidis GA, Weizman AV, Kashyap PC, Morgan RL (2020) AGA technical review on the role of probiotics in the management of gastrointestinal disorders. Gastroenterology 159:708-738.e4. 10.1053/j.gastro.2020.05.060 32531292 10.1053/j.gastro.2020.05.060 PMC8018518

[CR26] Quigley EMM, Fried M, Gwee K-A, Khalif I, Hungin P, Lindberg G, Abbas Z, Bhatia SJ, Schmulson M, Olano C, Le Mair A, Bustos Fernandez L (2015) World gastroenterology organisation global guidelines. Irritable bowel syndrome: a global perspective 10.1097/MCG.0000000000000653 27623513

[CR27] Rößler A (2023) EMA-Empfehlung: Neue Kontraindikationen für Pseudoephedrin. Pharmazeutische Zeitung Online

[CR28] Seifert R (2018) Basiswissen Pharmakologie. (Berlin; Heidelberg: Springer)

[CR29] Sempora Consulting GmbH (2024) SEMPORA Top Online Apotheken 2024

[CR30] Schnadower D, Tarr PI, Casper TC, Gorelick MH, Dean JM, O’Connell KJ, Mahajan P, Levine AC, Bhatt SR, Roskind CG, Powell EC, Rogers AJ, Vance C, Sapien RE, Olsen CS, Metheney M, Dickey VP, Hall-Moore C, Freedman SB (2018) Lactobacillus rhamnosus GG versus placebo for acute gastroenteritis in children. N Engl J Med 379:2002–2014. 10.1056/NEJMoa1802598 30462938 10.1056/NEJMoa1802598 PMC6358014

[CR31] Smith SM, Schroeder K, Fahey T (2014) Over-the-counter (OTC) medications for acute cough in children and adults in community settings. Cochrane Database Syst Rev 2014:001831. 10.1002/14651858.CD001831.pub5 10.1002/14651858.CD001831.pub5 PMC7061814 25420096

[CR32] Stey C, Steurer J, Bachmann S, Medici TC, Tramèr MR (2000) The effect of oral N-acetylcysteine in chronic bronchitis: a quantitative systematic review. Eur Respir J 16:253–262. 10.1034/j.1399-3003.2000.16b12.x 10968500 10.1034/j.1399-3003.2000.16b12.x

[CR33] Stuck BA, Popert U, Beule A, Jobst D, Klimek L, Laudien M, Lell M, Vogl TJ, Deutsche Gesellschaft für Allgemeinmedizin und Familienmedizin e.V., and Deutsche Gesellschaft für Hals-Nasen-Ohren-Heilkunde, Kopf- und Hals-Chirurgie e.V. (2017) S2k-Leitlinie Rhinosinusitis 10.1007/s00106-019-0695-6 31175378

[CR34] Szajewska H, Guarino A, Hojsak I, Indrio F, Kolacek S, Orel R, Salvatore S, Shamir R, van Goudoever JB, Vandenplas Y, Weizman Z, Zalewski BM (2020) Use of probiotics for the management of acute gastroenteritis in children: an update. J Pediatr Gastroenterol Nutr 71:261–269. 10.1097/MPG.0000000000002751 32349041 10.1097/MPG.0000000000002751

[CR35] Voigt K, Borchers P, Brosse F, Chenot J-F, Haasenritter J, Kötter T, Muche-Borowski C, Schübel J (2023) Innovationsausschuss des Gemeinsamen Bundesausschusses (G-BA) fördert neue und alte DEGAM-Leitlinien. Z Allg Med 99:80–85. 10.1007/s44266-022-00015-x

[CR36] Wade AG, Morris C, Shephard A, Crawford GM, Goulder MA (2011) A multicentre, randomised, double-blind, single-dose study assessing the efficacy of AMC/DCBA Warm lozenge or AMC/DCBA Cool lozenge in the relief of acute sore throat. BMC Fam Pract 12:6. 10.1186/1471-2296-12-6 21332976 10.1186/1471-2296-12-6 PMC3050701

[CR37] Wöckel A, Kurzeder C, Geyer V, Novasphenny I, Wolters R, Wischnewsky M, Kreienberg R, Varga D (2010) Effects of guideline adherence in primary breast cancer–a 5-year multi-center cohort study of 3976 patients. Breast 19:120–127. 10.1016/j.breast.2009.12.006 20117932 10.1016/j.breast.2009.12.006

[CR38] Zuberbier T, Abdul Latiff AH, Abuzakouk M, Aquilina S, Asero R, Baker D, Ballmer-Weber B, Bangert C, Ben-Shoshan M, Bernstein JA, Bindslev-Jensen C, Brockow K, Brzoza Z, Chong Neto HJ, Church MK, Criado PR, Danilycheva IV, Dressler C, Ensina LF, Fonacier L, Gaskins M, Gáspár K, Gelincik A, Giménez-Arnau A, Godse K et al (2022) The international EAACI/GA^2^LEN/EuroGuiDerm/APAAACI guideline for the definition, classification, diagnosis, and management of urticaria. Allergy 77:734–766. 10.1111/all.15090 34536239 10.1111/all.15090

