# Supplementary material for: Substantial non-compliance of online pharmacy catalogues with guidelines
Source: Naunyn Schmiedebergs Arch Pharmacol. 2024 Nov 12;398(5):5195–211. doi: 10.1007/s00210-024-03571-0 (PMC11985653; doi:10.1007/s00210-024-03571-0)
Supplement: Supplementary file 1 — Supplementary file1 (DOCX 81 KB) [file 210_2024_3571_MOESM1_ESM.docx]

**Supplemental data**

**Substantial non-compliance of online pharmacy catalogues with guidelines of the Association of the Scientific Medical Societies in Germany (AWMF)**

**Lara Barlage and Roland Seifert**


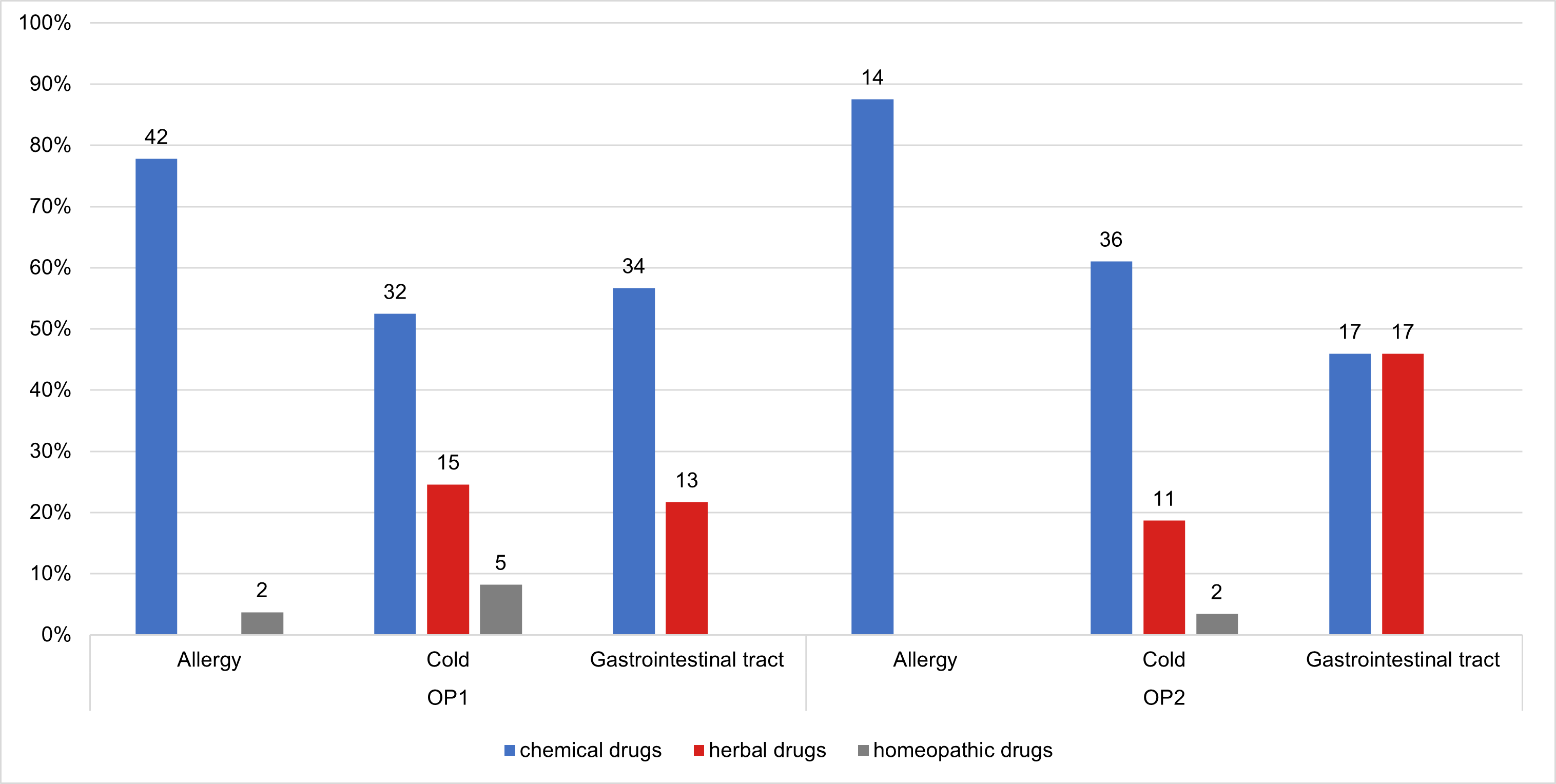


Figure S1: Illustration of the proportion per category of active pharmaceutical ingredients in the product catalogues of the online pharmacies OP1 and OP2; chemical pharmaceuticals in blue, herbal pharmaceuticals in red and homeopathic pharmaceuticals in grey

**Table S1:** Overview of the AWMF and international guidelines

| Indication | AWMF-Title | Link (last accessed 23 July) | International guideline | Link (last accessed 23 July) |
| --- | --- | --- | --- | --- |
| Urticaria | S3-Leitlinie Klassifikation, Diagnostik und Therapie der Urtikaria | https://register.awmf.org/de/leitlinien/detail/013-028 | The international EAACI/GA²LEN/EuroGuiDerm/APAAACI guideline for the definition, classification, diagnosis, and management of urticaria | https://onlinelibrary.wiley.com/doi/full/10.1111/all.15090 |
| Gastrointestinal Infections/ Acute Diarrhea | Update S2k-Leitlinie Gastrointestinale Infektionen | https://register.awmf.org/de/leitlinien/detail/021-024 | *WGO Acute Diarrhea* | *https://www.worldgastroenterology.org/guidelines/acute-diarrhea* |
| Irritable Bowel Syndrome | Update S3-Leitlinie Reizdarmsyndrom: Definition, Pathophysiologie,  Diagnostik und Therapie. | https://register.awmf.org/de/leitlinien/detail/021-016 | WGO Practice Guideline  Irritable Bowel Syndrome (IBS) | https://www.worldgastroenterology.org/guidelines/irritable-bowel-syndrome-ibs |
| GERD | S2k-Leitlinie Gastroösophageale Refluxkrankheit und eosinophile  Ösophagitis | https://register.awmf.org/de/leitlinien/detail/021-013 | WGO Practice Guideline  Gastroesophageal Reflux Disease (GERD) | https://www.worldgastroenterology.org/guidelines/gastroesophageal-reflux-disease |
| Constipation | Aktualisierte S2k-Leitlinie chronische Obstipation | https://register.awmf.org/de/leitlinien/detail/021-019 | WGO Practice Guideline  Constipation; | https://www.worldgastroenterology.org/guidelines/constipation |
| haemorrhoidal disease | S3-Leitlinie - Hämorrhoidalleiden | https://register.awmf.org/de/leitlinien/detail/081-007 | European Society of ColoProctology: guideline for  haemorrhoidal disease | https://aecp-es.org/images/site/documentos/GUIAS/ESCP_haemorrhoids20.pdf |
| Cough | S3-Leitlinie Akuter und chronischer Husten | https://register.awmf.org/de/leitlinien/detail/053-013 | Cough (acute): antimicrobial prescribing (NICE) | https://www.nice.org.uk/guidance/ng120/chapter/Recommendations#treatment |
| Sore throat | S3-Leitlinie Halsschmerzen | https://register.awmf.org/de/leitlinien/detail/053-010 | Sore throat (acute): antimicrobial prescribing (NICE) | https://www.nice.org.uk/guidance/ng84/chapter/Recommendations#managing-acute-sore-throat |
| Rhinosinusitis | S2k-Leitlinie Rhinosinusitis | https://register.awmf.org/de/leitlinien/detail/053-012 | International consensus statement on allergy and  rhinology: rhinosinusitis 2021 | https://onlinelibrary.wiley.com/doi/10.1002/alr.22741 |
